# CO_2_-laser-induced carbonization of calcium chloride-treated chitin nanopaper for applications in solar thermal heating†

**DOI:** 10.1039/d3ra03373b

**Published:** 2023-06-12

**Authors:** Thanakorn Yeamsuksawat, Luting Zhu, Takaaki Kasuga, Masaya Nogi, Hirotaka Koga

**Affiliations:** a SANKEN (The Institute of Scientific and Industrial Research), Osaka University 8-1 Mihogaoka Ibaraki Osaka 567-0047 Japan hkoga@eco.sanken.osaka-u.ac.jp +81-6-6879-8444 +81-6-6879-8442

## Abstract

Remarkable progress has been made in the development of carbonized chitin nanofiber materials for various functional applications, including solar thermal heating, owing to their N- and O-doped carbon structures and sustainable nature. Carbonization is a fascinating process for the functionalization of chitin nanofiber materials. However, conventional carbonization techniques require harmful reagents, high-temperature treatment, and time-consuming processes. Although CO_2_ laser irradiation has progressed as a facile and second-scale high-speed carbonization process, CO_2_-laser-carbonized chitin nanofiber materials and their applications have not yet been explored. Herein, we demonstrate the CO_2_-laser-induced carbonization of chitin nanofiber paper (denoted as chitin nanopaper) and investigate the solar thermal heating performance of the CO_2_-laser-carbonized chitin nanopaper. While the original chitin nanopaper was inevitably burned out by CO_2_ laser irradiation, CO_2_-laser-induced carbonization of the chitin nanopaper was achieved by pretreatment with calcium chloride as a combustion inhibitor. The CO_2_-laser-carbonized chitin nanopaper exhibits excellent solar thermal heating performance; its equilibrium surface temperature under 1 sun irradiation is 77.7 °C, which is higher than those of the commercial nanocarbon films and the conventionally carbonized bionanofiber papers. This study paves the way for the high-speed fabrication of carbonized chitin nanofiber materials and their application in solar thermal heating toward the effective utilization of solar energy as heat.

## Abstract 2

The second-scale CO_2_-laser-induced carbonization of chitin nanopaper was achieved by CaCl_2_ pretreatment. The as-carbonized chitin nanopaper exhibited excellent solar thermal heating performance for the effective utilization of solar energy as heat.

## Graphical abstract

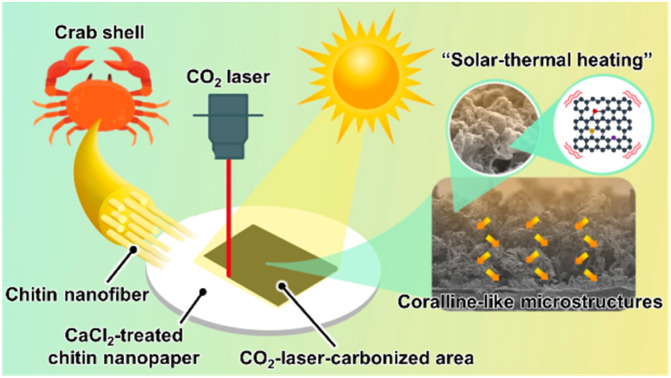

## Introduction

Carbonized biomass materials have garnered increasing attention owing to their light weight, large specific surface areas, unique electrical properties, high thermal stability, and sustainable nature.^1^ The functional design of carbonized biomass materials has been actively investigated for various applications, such as adsorption/separation,^2,3^ sensing,^4^ energy generation,^4^ and storage.^5–7^ Among various biomass materials, chitin (β-(1→4)-linked *N*-acetyl anhydroglucosamine), which is mainly derived from marine creatures such as crabs and shrimps,^8^ has been regarded as a promising material for functional design by carbonization. For instance, carbonized chitin nanofiber materials can provide multiple functions, including sensing,^8,9^ energy storage for supercapacitors,^8–10^ and microwave absorption^11^ by tailoring their N- and O-doped carbon structures, as well as their porous nano/microstructures.

One of the emerging applications of carbonized biomass materials is solar thermal heating.^12–16^ Solar thermal heating is a phenomenon to absorb and convert solar light into thermal energy, which can be achieved using photothermal materials. Carbonized biomass materials afford broadband light absorption, which is beneficial for absorbing solar light in the wavelength range of 300–2500 nm (ASTM G173-03, Air Mass 1.5 Global spectrum (AM1.5G)).^17^ The absorption-wavelength range is wider than those of other photothermal materials, such as plasmonic nanomaterials (approximately 300–1000 nm) and oxide semiconductors (approximately 300–1500 nm).^18^ Accordingly, carbonized biomass materials are expected to be sustainable and high-performance photothermal materials. Thus, solar thermal heating by carbonized biomass materials has become a major center of attraction toward the effective use of renewable solar energy and is used in various applications, including photothermal catalysis,^19^ solar steam generation,^20^ wastewater purification,^21^ desalination,^22^ and thermoelectric generation.^15^

Carbonization is essential for the fabrication of carbonized biomass materials. Biomass materials have been traditionally carbonized by acid,^23,24^ hydrothermal,^3,5,25,26^ and high-temperature treatments.^2–16^ Although these traditional carbonization treatments require harmful reagents^23,24^ or time-consuming processes,^2–16,25,26^ CO_2_-laser irradiation treatment has been progressing as a facile and second-scale fast carbonization process.^27–32^ The CO_2_-laser-induced carbonization has been first reported for polyimide,^27^ and thereafter actively applied for various organic polymer materials including biomass materials.^28–32^ Upon CO_2_ laser irradiation, the organic polymer materials reach a high temperature, causing their chemical bonds such as C–O and C=O to break and rearrange to form a graphitic carbon structure.^27^ Recently, the CO_2_-laser-induced carbonization has been applied to a synthetic polybenzoxazine resin film to fabricate a graphitic carbon with forest-like morphologies, providing excellent solar thermal heating properties.^33^ To the best of our knowledge, however, the CO_2_-laser-induced carbonization of biomass materials for solar thermal heating applications has been unexplored.

In our previous study, the high solar-thermal heating performance of a high-temperature (400 °C) carbonized chitin nanofiber paper (denoted as chitin nanopaper) was reported.^16^ Herein, we showed the CO_2_-laser-induced carbonization of chitin nanopaper and further evaluated the CO_2_-laser-carbonized chitin nanopaper as a photothermal material for solar thermal heating. The chitin nanopaper inevitably burned out during CO_2_ laser irradiation, indicating the difficulty of its CO_2_-laser-induced carbonization. To inhibit combustion, the chitin nanopaper was pretreated with calcium chloride (CaCl_2_). CaCl_2_-treated chitin nanopaper was successfully carbonized using seconds-scale CO_2_-laser irradiation process. Excellent solar thermal heating performance of the CO_2_-laser-carbonized chitin nanopaper was also demonstrated.

## Results and discussion

### CaCl_2_ treatment of chitin nanopaper for CO_2_-laser-induced carbonization

**Fig. 1:**
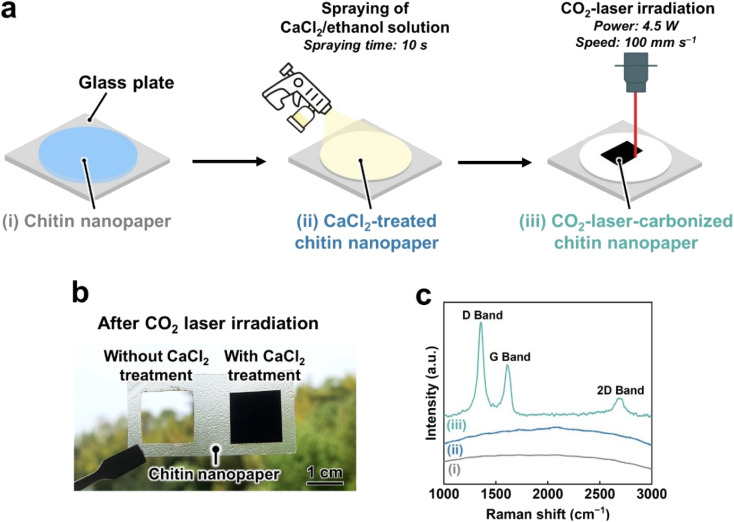
CaCl_2_ treatment and CO_2_-laser-induced carbonization of chitin nanopaper. (a) Procedure schematic, (b) optical image of chitin nanopaper with or without the CaCl_2_ treatment after CO_2_ laser irradiation, and (c) Raman spectra of the (i) original chitin nanopaper, (ii) CaCl_2_-treated chitin nanopaper, and (iii) CO_2_-laser-carbonized chitin nanopaper.

CO_2_-laser-induced carbonization of the chitin nanopaper was performed according to the workflow shown in Fig. 1a. Chitin nanopapers were prepared from a crab-shell-derived chitin nanofiber/water dispersion by suction filtration and subsequent hot-press drying. When the CO_2_ laser was irradiated with the chitin nanopaper without any pretreatment, it completely burned out, even under an inert nitrogen atmosphere (Fig. 1b, left), indicating that the chitin nanopaper was difficult to carbonize by CO_2_ laser irradiation. Such difficulties have been frequently observed for various biomass materials owing to their low thermal stability; they have been subjected to fire-retardant treatments such as additive mixing^28,31,32^ and chemical modification.^29,30^ To overcome this problem, in this study, CaCl_2_, which has been used as a combustion inhibitor,^34–37^ was introduced into chitin nanopaper. As depicted in Fig. 1a, chitin nanopaper (approximately 400 mg) with a diameter and thickness of approximately 70 mm and 100 μm, respectively, was sprayed with an approximately 25 wt% CaCl_2_/ethanol solution for 10 s. After drying, the CaCl_2_ content of the chitin nanopaper was approximately 78 mg. The CaCl_2_-treated chitin nanopaper with a thickness of approximately 105 μm was thereafter irradiated with a CO_2_ laser at a power and speed of 4.5 W and 100 mm s^−1^, respectively. The CO_2_-laser-irradiated area on the CaCl_2_-treated chitin nanopaper turned black (Fig. 1b, right). In the Raman spectra, three characteristic peaks, namely, the D band (approximately 1350 cm^−1^),^38^ G band (approximately 1600 cm^−1^),^38^ and 2D band (approximately 2680 cm^−1^)^39^ were confirmed in the CO_2_-laser-irradiated area, whereas these peaks were not observed in the original and CaCl_2_-treated chitin nanopapers before CO_2_ laser irradiation (Fig. 1c). The D, G, and 2D bands are reportedly ascribed to defective carbon structures,^38^ graphitic sp^2^-hybridized carbon structures,^38^ and stacking of graphitic carbon structures,^39^ respectively. These results indicated that the CO_2_-laser-induced carbonization of the chitin nanopaper was successfully achieved by CaCl_2_ pretreatment. The CO_2_-laser-induced carbonization process of the CaCl_2_-treated chitin nanopaper (carbonization area: 1.5 cm × 1.5 cm) was completed in approximately 32 s, which was much faster than the conventional hours-scale carbonization processes of chitin materials such as hydrothermal^25^ and high-temperature^7–11,16^ treatments.

### Chemical structures of CO_2_-laser-carbonized chitin nanopaper

**Fig. 2:**
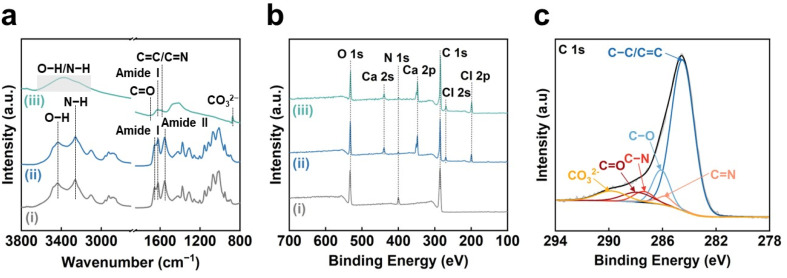
Chemical structures of the CO_2_-laser-carbonized chitin nanopaper. (a) FT-IR and (b) wide XPS spectra of the (i) original chitin nanopaper, (ii) CaCl_2_-treated chitin nanopaper, and (iii) CO_2_-laser-carbonized chitin nanopaper. (c) C 1s XPS spectrum of the CO_2_-laser-carbonized chitin nanopaper.

**Fig. 3:**
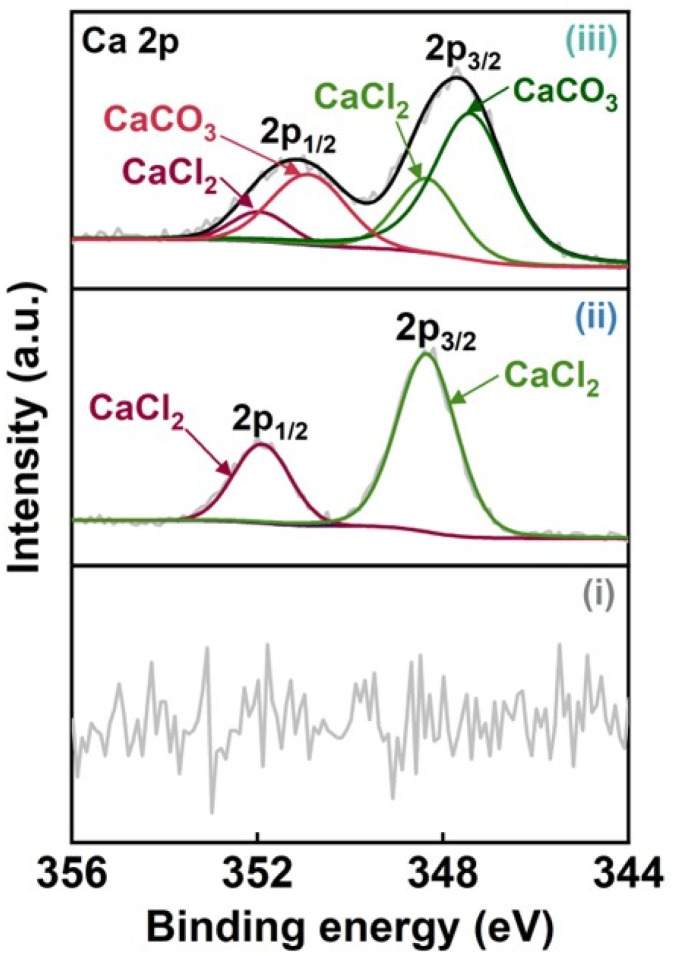
Ca 2p XPS spectra of the (i) original chitin nanopaper, (ii) CaCl_2_-treated chitin nanopaper, and (iii) CO_2_-laser-carbonized chitin nanopaper.

The chemical structures of the CO_2_-laser-carbonized chitin nanopaper were further analyzed by Fourier-transform infrared (FT-IR) spectroscopy and X-ray photoelectron spectroscopy (XPS) (Fig. 2). In the FT-IR spectra (Fig. 2a), the original chitin nanopaper exhibited characteristic peaks corresponding to the O–H groups (3450 cm^−1^),^40^ N–H groups (3260 cm^−1^),^40^ amide I region (1660 and 1620 cm^−1^),^41^ and amide II region (1560 cm^−1^),^41^ which are derived from α-chitin.^41^ The CaCl_2_-treated chitin nanopaper exhibited similar spectra with the original chitin nanopaper, indicating that the original chemical structure of chitin nanopaper remained unchanged after the CaCl_2_ treatment. The original chemical structure changed significantly after CO_2_ laser irradiation. The CO_2_-laser-carbonized chitin nanopaper exhibited the formation of C=C and C=N (1580 cm^−1^),^42^ and C=O groups (1700 cm^−1^),^42^ while retaining the C=O groups of the acetyl unit on amide I (1620 cm^−1^),^43^ and O–H and N–H groups. Notably, the characteristic peak of carbonate (CO_3_^2−^) appeared at 875 cm^−1^, suggesting the formation of calcium carbonate (CaCO_3_)^44^ after the CO_2_ laser carbonization. The wide XPS spectrum of the CO_2_-laser-carbonized chitin nanopaper indicated the presence of C, O, N, Ca, and Cl (Fig. 2b). The C 1s XPS spectrum of the CO_2_-laser-carbonized chitin nanopaper could be divided into six peaks at 284.6, 285.8, 286.1, 287.4, 287.8, and 290.0 eV, which are attributed to C–C or C=C, C=N, C–O, C–N, C=O, and CO_3_^2−^, respectively^45–48^ (Fig. 2c). From the results of Raman, FT-IR, and XPS analyses (Fig. 1c and 2), it was indicated that the CO_2_-laser-carbonized chitin nanopaper has N- and O-doped defective carbon structures, as reported for the high-temperature-carbonized chitin nanopaper.^16^ While the Ca 2p XPS spectrum of the CaCl_2_-treated chitin nanopaper exhibited characteristic peaks at 348.3 eV for 2p_3/2_ and 351.8 eV for 2p_1/2_ corresponding to CaCl_2_,^49^ that of the CO_2_-laser-carbonized chitin nanopaper exhibited additional peaks at 347.4 eV for 2p_3/2_ and 350.9 eV for 2p_1/2_ corresponding to CaCO_3_ (ref. 50 and 51) (Fig. 3). The formation of CaCO_3_ upon the CO_2_-laser-induced carbonization is due to the presence of CaCl_2_, CO_2_, and H_2_O,^35^ where CO_2_ and H_2_O can be generated by the combustion chain reaction of chitin upon its carbonization.^52^

The CO_2_-laser-induced carbonization of the CaCl_2_-treated chitin nanopaper can be ascribed to the generation of thermal energy (high temperatures) through the photothermal effect derived from the lattice vibrations of chitin molecules, as reported for polyimide.^27^ Although the original chitin nanopaper burned out upon CO_2_ laser irradiation, such combustion could be suppressed in the presence of CaCl_2_ (Fig. 1c). The combustion-inhibiting effect of CaCl_2_ can be explained as follows. During carbonization of polymeric materials, reactive radicals, such as ˙OH and ˙H, are continuously generated, promoting the combustion reaction of polymeric materials.^53^ The Ca and Cl ions can reportedly scavenge these radicals,^34–37^ thereby inhibiting the combustion of polymeric materials. Hence, the CO_2_-laser-induced carbonization of chitin nanopaper was successfully achieved in the presence of CaCl_2_.

### Morphologies of CO_2_-laser-carbonized chitin nanopaper

**Fig. 4:**
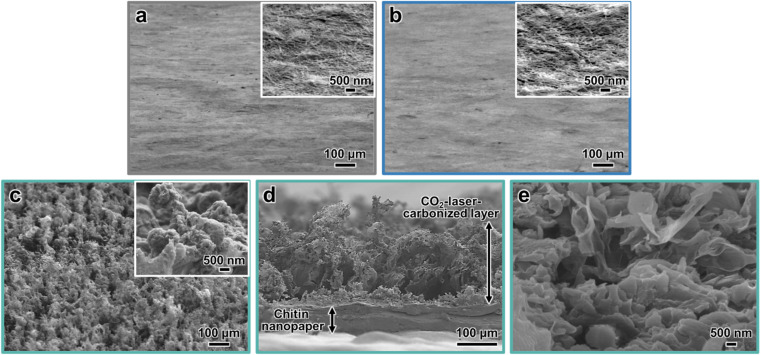
Morphologies of the CO_2_-laser-carbonized chitin nanopaper. Oblique-view FE-SEM images of the (a) original chitin nanopaper, (b) CaCl_2_-treated chitin nanopaper, and (c) CO_2_-laser-carbonized chitin nanopaper. (d and e) Cross-section FE-SEM image of the CO_2_-laser-carbonized chitin nanopaper.

The morphologies of the chitin nanopapers were observed by field-emission scanning electron microscopy (FE-SEM) (Fig. 4). The original chitin nanopaper had smooth surfaces derived from the densely packed chitin nanofibers (Fig. 4a). Smooth surface structures were maintained after CaCl_2_ treatment (Fig. 4b). After CO_2_-laser-induced carbonization, the surface structures became rough and porous (Fig. 4c), forming coralline-like microstructures, as observed in the CO_2_-laser-carbonized polybenzoxazine resin film^33^ (inset of Fig. 4c–e). The thicknesses of the original and CaCl_2_-treated chitin nanopapers were approximately 100 and 105 μm, respectively. The CO_2_-laser-carbonized chitin nanopaper had two layers comprising a chitin nanopaper and a CO_2_-laser-carbonized area with thicknesses of approximately 70 and 160 μm, respectively (Fig. 4d and S1†). These results suggest that approximately 35 μm of the surface layer of the CaCl_2_-treated chitin nanopaper was carbonized by CO_2_ laser irradiation to form a carbonized layer of approximately 160 μm. Such a relatively thick CO_2_-laser-carbonized layer was formed owing to a “bombing” effect;^54^ byproduct gases were released quickly upon the rapid CO_2_-laser-induced carbonization to form a porous and thick carbonized layer.

### Solar absorption and solar thermal heating performances of CO_2_-laser-carbonized chitin nanopaper

**Fig. 5:**
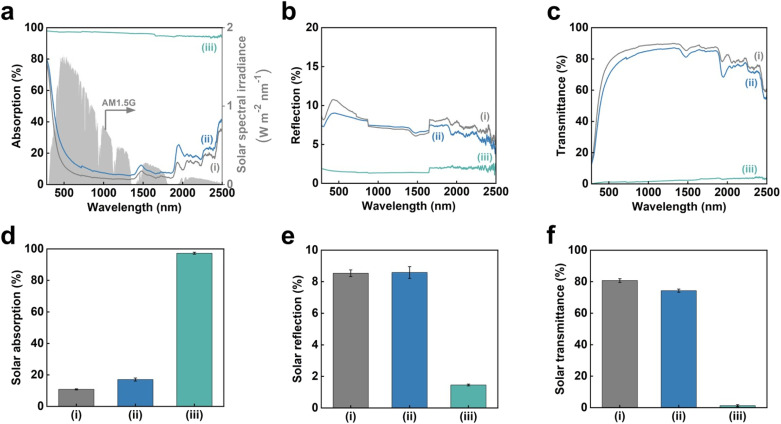
Solar absorption of the CO_2_-laser-carbonized chitin nanopaper. (a) Solar spectral irradiance (AM1.5G) and UV-vis-NIR absorption, (b) reflection, (c) transmittance spectra, solar (d) absorption, (e) reflection, and (f) transmittance of the (i) original chitin nanopaper, (ii) CaCl_2_-treated chitin nanopaper, and (iii) CO_2_-laser-carbonized chitin nanopaper.

CO_2_-laser-carbonized chitin nanopaper was applied as a photothermal material for solar thermal heating. The solar thermal heating performance of photothermal materials is determined by their capacity to absorb and convert solar light into heat.^18^ Accordingly, the light absorption properties of the CO_2_-laser-carbonized chitin nanopaper were first evaluated. Fig. 5a–c shows the ultraviolet-visible-near infrared (UV-vis-NIR) absorption, reflection, and transmission properties of the original chitin nanopaper, CaCl_2_-treated chitin nanopaper, and CO_2_-laser-carbonized chitin nanopaper. The original and CaCl_2_-treated chitin nanopapers exhibited poor light absorption owing to their high light reflection and transmission. In contrast, the CO_2_-laser-carbonized chitin nanopaper achieved much higher light absorption by suppressing reflection and transmission; the light absorption ranged from approximately 98 to 94% in the wavelength range of solar light from 300 to 2500 nm (AM1.5G).

For a clearer discussion, the solar absorption was estimated from the UV-vis-NIR absorption spectra, according to the following equation:^55^
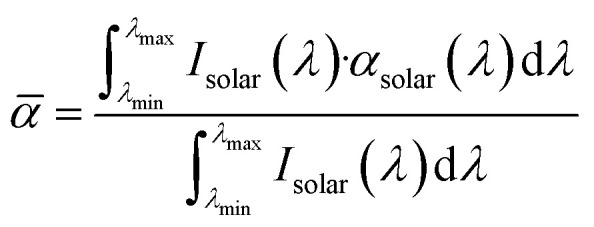
where *ᾱ* denotes the solar absorption (%), *λ* denotes the wavelength (nm), *λ*_min_ and *λ*_max_ are 300 and 2500 nm, respectively, *I*_solar_(*λ*) denotes the AM1.5G solar spectral irradiance at *λ*, and *α*_solar_(*λ*) denotes the light absorption (%) at *λ*. The solar reflection and transmittance were estimated in a similar manner. Fig. 5d–f displays the estimated solar absorption, reflection, and transmittance values of the original chitin nanopaper, CaCl_2_-treated chitin nanopaper, and CO_2_-laser-carbonized chitin nanopaper. The solar absorption, reflection, and transmittance of the original chitin nanopaper were 10.8, 8.5, and 80.7%, respectively, whereas those of the CaCl_2_-treated chitin nanopaper were 17.1, 8.6, and 74.3%, respectively. These results indicate that the solar absorption properties of the chitin nanopaper did not change significantly after CaCl_2_ treatment, because the original chemical structure of chitin nanopaper remained unchanged after the CaCl_2_ treatment (Fig. 2a) and the CaCl_2_ itself exhibited low solar absorption. Notably, solar absorption was drastically improved to 97.3% by CO_2_-laser-induced carbonization, while solar reflection and transmission decreased to 1.5 and 1.2%, respectively. Solar absorption of the CO_2_-laser-carbonized chitin nanopaper is owing to (1) its N- and O-doped carbon structures and (2) its coralline-like porous microstructure, as follows: (1) while the original and CaCl_2_-treated chitin nanopapers had high optical bandgap values (approximately 5 eV), the CO_2_-laser-carbonized chitin nanopaper had a much lower optical bandgap (approximately 0.5 eV) (Fig. S2†), facilitating light absorption at lower energies (*i.e.*, longer wavelengths). The original and CaCl_2_-treated chitin nanopapers possessed a wide σ–σ* bandgap derived from the sp^3^-hybridized carbon structure of chitin. The CO_2_-laser-carbonized chitin nanopaper with graphitic sp^2^-hybridized carbon structures (Fig. 1c) has π–π* bandgap, which is located within the σ–σ* bandgap.^56^ Moreover, the CO_2_-laser-carbonized chitin nanopaper contained the N- and O-containing functional groups (n-orbital) (Fig. 2), where the *n* energy level is located within the π–π* bandgap^57^ and, thus, can further decrease the optical bandgap.^16^ (2) The coralline-like porous microstructures (Fig. 4c and d) can also enhance light absorption^33^ by facilitating multiple light reflections inside the CO_2_-laser-carbonized chitin nanopaper (*i.e.*, by suppressing the light reflection to its outer surface), as in a light confinement effect.^58^

**Fig. 6:**
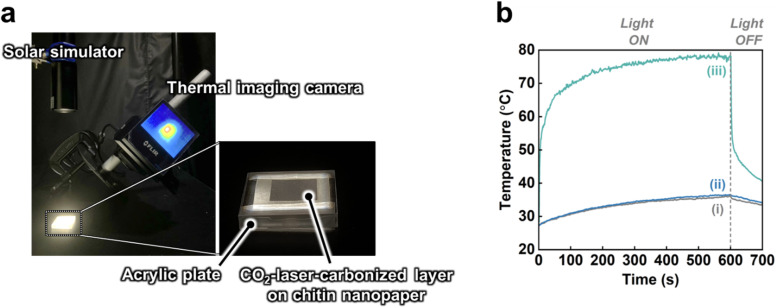
Solar thermal heating performance of the CO_2_-laser-carbonized chitin nanopaper. (a) Experimental setup for the surface temperature measurement during 1 sun irradiation by a solar simulator, (b) surface temperature *versus* 1-sun irradiation time of the (i) original chitin nanopaper, (ii) CaCl_2_-treated chitin nanopaper, and (iii) CO_2_-laser-carbonized chitin nanopaper.

Subsequently, the solar thermal heating performance of the CO_2_-laser-carbonized chitin nanopaper was investigated. Change in the surface temperature during simulated solar irradiation (light intensity: 1.0 kW m^−2^ (1 sun), AM1.5G) was monitored using a thermal imaging camera (Fig. 6a). As shown in Fig. 6b, the surface temperature of the CO_2_-laser-carbonized chitin nanopaper sharply rose upon 1 sun irradiation and reached up to 77.7 ± 0.99 °C within 600 s, which was much higher than those of the original and CaCl_2_-treated chitin nanopapers (36.1 ± 0.35 and 36.1 ± 0.19 °C, respectively). The original and CaCl_2_-treated chitin nanopapers exhibited poor solar thermal heating performances, due to the low solar absorption of the chitin nanopaper and CaCl_2_ (Fig. 5d). The high solar thermal heating performance of the CO_2_-laser-carbonized chitin nanopaper was attributed to its effective solar absorption (97.3%). The CaCl_2_ pretreatment time (*i.e.*, spraying time of the 25 wt% CaCl_2_/ethanol solution) and CO_2_ laser power affected the solar thermal heating performance of the CO_2_-laser-carbonized chitin nanopaper; it exhibited the highest surface temperature at a CaCl_2_ pretreatment time of 10 s (addition amount of CaCl_2_ for approximately 400 mg and 38.5 cm^2^ of the chitin nanopaper: approximately 78 mg) and a CO_2_ laser power of 4.5 W. It was suggested that CaCl_2_ pretreatment for 10 s can balance the combustion inhibition during carbonization and the surface exposure of the carbonized layer to solar light, while a laser power of 4.5 W can balance the progress of carbonization and the suppression of combustion (see Fig. S3† for more details). The equilibrium surface temperature of the CO_2_-laser-carbonized chitin nanopaper upon 1 sun irradiation (77.7 ± 0.99 °C) was higher even than those of the commercial carbon nanotube (55.0 °C)^15^ and graphene oxide (69.4 °C)^15^ films, high-temperature carbonized cellulose nanopaper (73.9 °C),^15^ and high-temperature carbonized chitin nanopaper (75.9 °C).^16^ Thus, the CO_2_-laser-carbonized chitin nanopaper is expected to be a high-performance photothermal material for solar thermal heating.

## Conclusion

In conclusion, the fast carbonization of chitin nanopaper was successfully achieved by CO_2_ laser irradiation. Pretreatment of chitin nanopaper with CaCl_2_ as a combustion inhibitor is the key to achieve the CO_2_-laser-induced carbonization. While conventional hydrothermal and high-temperature carbonization require time-consuming hour-scale processes, CO_2_-laser-induced carbonization can be performed on a second timescale. The CO_2_-laser-carbonized chitin nanopaper has N- and O-doped carbon structures and coralline-like microstructures, affording a high solar absorption of up to 97.3%. Furthermore, the CO_2_-laser-carbonized chitin nanopaper provided the excellent solar thermal heating performance (equilibrium surface temperature under 1 sun irradiation: 77.7 °C). Further functional designs and applications of the CO_2_-laser-carbonized chitin nanopaper can be expected. This study opens the door for the facile fabrication of carbonized biomass materials as sustainable photothermal materials toward the effective utilization of renewable solar energy as thermal energy.

## Experimental section

### Materials

A chitin nanofiber/water dispersion (2 wt%, SFo-20002) was supplied by Sugino Machine, Ltd (Namerikawa, Japan). CaCl_2_ (>95.0% purity) and ethanol (>99.5% purity) were obtained from Nacalai Tesque, Inc. (Kyoto, Japan).

### Preparation of chitin nanopaper

A chitin nanofiber/water dispersion (0.2 wt%, 200 mL) was dewatered by suction filtration through a hydrophilic polytetrafluoroethylene membrane with a pore diameter of 0.2 μm (H020A090C, Advantec Toyo Kaisha, Ltd, Tokyo, Japan). The wet sheet obtained was peeled from the membrane, placed on a hydrophobic glass plate, covered with a #400 stainless-steel wire mesh (CLV-SUS304M-48, Kurubaa Co., Ltd, Toyohashi, Japan), and sandwiched between paper towels (Kimtowel White, Nippon Paper Crecia Co., Ltd., Tokyo, Japan). Finally, the resulting assembly was dried by hot pressing at 110 °C and 0.35 MPa for 25 min (AYSR-5, Shinto Metal Industries, Ltd, Osaka, Japan) to prepare chitin nanopaper with a diameter and thickness of approximately 70 mm and 100 μm, respectively.

### Treatment of chitin nanopaper with CaCl_2_

Prior to the CaCl_2_ treatment, the as-prepared chitin nanopaper was fixed on a glass plate. Subsequently, a CaCl_2_/ethanol solution (CaCl_2_ content: approximately 25 wt%) was sprayed onto the surface of the chitin nanopaper for 5, 10, or 20 s using a spray machine (Handheld Nano Spray K5, Shenzhen NOYAFA Electronic Co., Ltd, Shenzhen, China). The distance between the spray jet and chitin nanopaper was set to approximately 10 cm while the spray jet was held at a 45° to the chitin nanopaper. The as-sprayed chitin nanopaper was stored in a fume hood under ambient conditions for at least 2 h. The CaCl_2_ content of the resulting chitin nanopaper was calculated by subtracting the dry weight of the CaCl_2_-treated chitin nanopaper from that of the chitin nanopaper.

### CO_2_-laser-induced carbonization of the CaCl_2_-treated chitin nanopaper

The CaCl_2_-treated chitin nanopaper on a glass plate was irradiated using a CO_2_ laser with a wavelength of 10.2 μm and a laser spot diameter of 300 μm (CO_2_-MK-kit, Kokyo, Inc., Kyoto, Japan) under a nitrogen atmosphere to prepare the CO_2_-laser-carbonized chitin nanopaper. The focal distance was 160 mm. A computer-controlled laser program was used to control the laser pattern (square shape: 1.5 cm × 1.5 cm), scan speed (100 mm s^−1^), pulsed laser frequency (25 kHz), and laser power (1.5, 3.0, 4.5, or 6.0 W) for a single irradiation loop.

### Solar thermal heating performance testing

Solar thermal heating performances were measured in a dark room, according to our previous studies.^15,16,59^ The emissivity of each sample was first measured using a black tape with a known emissivity of 0.95 (HB250, OPTEX Co., Ltd, Otsu, Japan) as a reference. In brief, the sample and black tape were heated to 75 °C by an SBX-303 temperature-controller (Sakaguchi E.H. VOC Corp., Tokyo, Japan). The emissivity of the sample was evaluated using an FLIR ETS320 thermal imaging camera (FLIR Systems. Inc., Wilsonville, USA) by adjusting the temperature based on the reference temperature of black tape. After emissivity calibration, the solar thermal heating performance of each sample was evaluated by measuring its surface temperature under 1 sun irradiation as follows: the 2.5 cm × 2.5 cm of chitin nanopaper, CaCl_2_-treated chitin nanopaper, or CO_2_-laser-carbonized chitin nanopaper (carbonized area: 1.5 cm × 1.5 cm) was placed on an acrylic plate (3 cm × 3 cm) with a central hole (0.7 cm × 0.7 cm). The change in surface temperature during simulated solar light irradiation (AM1.5G, light intensity: 1 sun, HAL-320W, Asahi Spectra Co., Ltd, Tokyo, Japan) was recorded by a thermal imaging camera (Fig. 6a). The solar light irradiation area was larger than that of the samples. The equilibrium surface temperature of the samples was estimated as the average surface temperature of approximately 850 plots during solar irradiation times of 500–600 s. This experiment was performed at approximately 25 °C and 50% relative humidity.

### Characterization

Chemical structures were analyzed by laser Raman spectroscopy (laser wavelength: 532 nm, RAMAN-touch VISNIR-OUN, Nanophoton Corp., Osaka, Japan), FT-IR/attenuated total reflection spectroscopy (KJP-05120S, PerkinElmer Japan Co., Ltd, Kanagawa, Japan), and XPS (JPS-9010, JEOL, Ltd, Tokyo, Japan) with a monochromatic AlKα X-ray source (1486.6 eV) at 15 kV and 20 mA. For FT-IR analysis of the CO_2_-laser-carbonized chitin nanopaper, the carbonized layer was scratched, recovered, and analyzed. The morphologies were observed by FE-SEM at an accelerating voltage of 2 kV (SU-8020, Hitachi High-Tech Science Corp., Tokyo, Japan). Prior to observation, platinum was sputtered onto the sample surfaces (E-1045 Ion Sputter, Hitachi High-Tech Science Corp., Tokyo, Japan). Optical absorption, reflection, and transmittance were measured using a UV-3600i Plus spectrometer with an ISR-603 integrating sphere attachment (Shimadzu Corp., Kyoto, Japan). The absorption (% *A*) was calculated from the transmittance (% *T*) and reflectance (% *R*) according to the following equation: % *A* = 100 − (% *T* + % *R*). The optical bandgap values of the original chitin nanopaper, CaCl_2_-treated chitin nanopaper, and CO_2_-laser-carbonized chitin nanopaper were estimated from the UV-vis-NIR absorbance spectra of their water dispersions, according to a previous study^4^ and Tauc's equation:^60^ (*αhν*)^1/*n*^ = *A*(*hν* − *E*_g_), where *α* denotes the absorbance, *hν* denotes the photon energy, *A* is a constant, and *E*_g_ denotes the optical bandgap. The parameter *n* is 1/2 and 2 for direct and indirect transitions, respectively. To estimate the optical bandgap, (*αhν*)^1/*n*^ was plotted against the photon energy (*hν*), and the linear region of the curve was extrapolated to the *x*-axis. The optical bandgap values of the chitin nanopaper and CaCl_2_-treated chitin nanopaper were estimated to be *n* = 1/2, whereas that of the CO_2_-laser-carbonized chitin nanopaper was estimated to be *n* = 2 because of its amorphous carbon structure.

## Author contributions

Conceptualization: T. Y. and H. K.; methodology: T. Y., L. Z., and H. K.; investigation, formal analysis, validation, data curation: T. Y.; visualization: T. Y. and H. K.; resources: T. K., M. N., and H. K.; project administration and funding acquisition: H. K.; supervision: M. N. and H. K.; writing—original draft preparation: T. Y.; writing—review and editing: L. Z., T. K., M. N., and H. K.

## Conflicts of interest

There are no conflicts to declare.

## Supplementary Material

RA-013-D3RA03373B-s001
