# Supplementary material for: CO2-laser-induced carbonization of calcium chloride-treated chitin nanopaper for applications in solar thermal heating
Source: RSC Adv. 2023 Jun 12;13(26):17556–64. doi: 10.1039/d3ra03373b (PMC10258603; doi:10.1039/d3ra03373b)
Supplement: RA-013-D3RA03373B-s001 [file RA-013-D3RA03373B-s001.pdf]

Electronic Supplementary Material (ESI) for RSC Advances.
This journal is © The Royal Society of Chemistry 2023

Supporting Information

CO_2-laser-induced carbonization of calcium chloride-treated chitin nanopaper for applications in solar thermal heating

Thanakorn Yeamsuksawat, Luting Zhu, Takaaki Kasuga, Masaya Nogi, and Hirotaka Koga[*]

SANKEN (The Institute of Scientific and Industrial Research), Osaka University, 8-1 Mihogaoka, Ibaraki, Osaka 567-0047, Japan

*Corresponding Author
Hirotaka Koga, E-mail: hkoga@eco.sanken.osaka-u.ac.jp

Supporting Figures

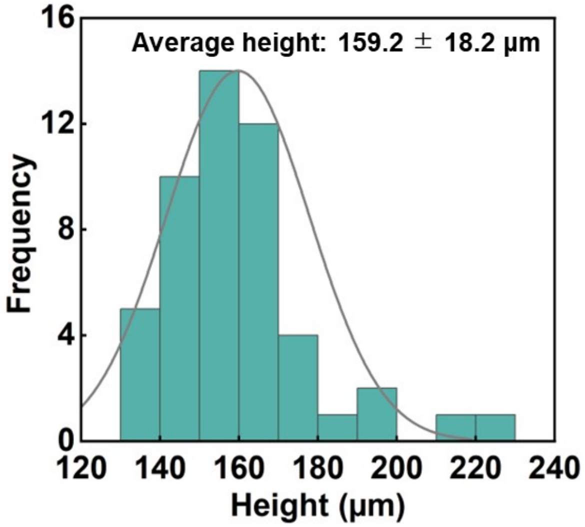

Figure S1. Height distribution histogram of the CO_2-laser-carbonized layer.

A height distribution histogram of the CO_2-laser-carbonized layer was obtained from the cross-sectional FE-SEM image of the CO_2-laser-carbonized chitin nanopaper (**Figure 4d**). The average height of the CO_2-laser-carbonized layer was calculated to be 159.2 ± 18.2 μm.

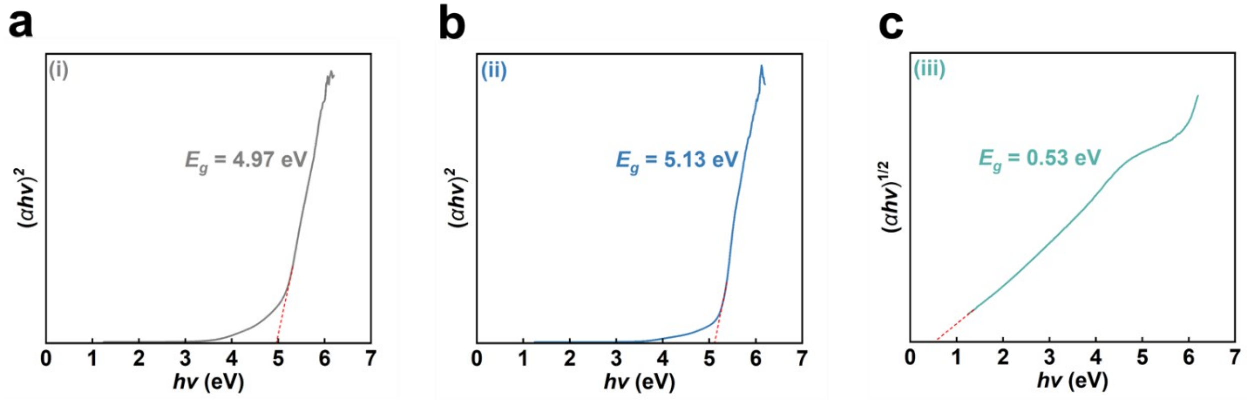

Figure S2. Tauc plots and estimated optical bandgap values of the (a) original chitin nanopaper, (b) $CaCl_2$-treated chitin nanopaper, and (c) CO_2-laser-carbonized chitin nanopaper.

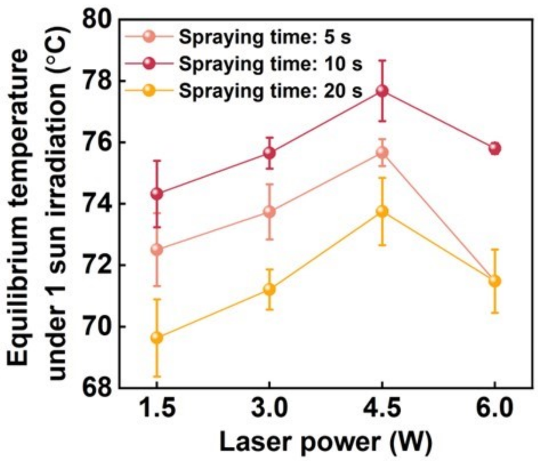

Figure S3. Equilibrium surface temperature under 1 sun irradiation of CO_2-laser-carbonized chitin nanopaper prepared with different spraying times of 25 wt% $CaCl_2$/ethanol solution and different CO_2 laser powers.

The effects of the $CaCl_2$ pretreatment time (spraying time of the 25 wt% $CaCl_2$/ethanol solution) and CO_2 laser power on the solar thermal heating performance of the CO_2-laser-carbonized chitin nanopaper were investigated (**Figure S3**). The CO_2-laser-carbonized chitin nanopaper prepared with a $CaCl_2$ pretreatment time of 10 s exhibited a higher surface temperature (i.e., higher solar thermal heating performance) than those prepared with $CaCl_2$ pretreatment times of 5 and 20 s, regardless of the examined laser power (1.5, 3.0, 4.5, or 6.0 W). Hence, the optimal $CaCl_2$ pretreatment time for providing the best solar thermal heating performance was determined to be 10 s. This is possibly because $CaCl_2$ pretreatment for 10 s can balance the combustion inhibition during carbonization and the surface exposure of the carbonized layer. Regardless of the $CaCl_2$ pretreatment time, the solar thermal heating performance of the CO_2-laser-carbonized chitin nanopaper was increased with increasing laser power from 1.5 to 4.5 W, while the solar thermal heating performance was decreased with increasing laser power from 4.5 to 6.0 W. Hence, the optimum laser power was considered as 4.5 W. This is possibly because a laser power of 4.5 W can balance the progress of carbonization and the suppression of combustion.
